# “Brave Men” and “Emotional Women”: A Theory-Guided Literature Review on Gender Bias in Health Care and Gendered Norms towards Patients with Chronic Pain

**DOI:** 10.1155/2018/6358624

**Published:** 2018-02-25

**Authors:** Anke Samulowitz, Ida Gremyr, Erik Eriksson, Gunnel Hensing

**Affiliations:** ^1^Epidemiology and Social Medicine, Institute of Medicine, Sahlgrenska Academy, University of Gothenburg, Göteborg SE-405 30, Sweden; ^2^Centre for Healthcare Improvment, Division of Service Management and Logistics, Department of Technology Management and Economics, Chalmers University of Technology, Göteborg SE-412 96, Sweden

## Abstract

**Background:**

Despite the large body of research on sex differences in pain, there is a lack of knowledge about the influence of gender in the patient-provider encounter. The purpose of this study was to review literature on gendered norms about men and women with pain and gender bias in the treatment of pain. The second aim was to analyze the results guided by the theoretical concepts of hegemonic masculinity and andronormativity.

**Methods:**

A literature search of databases was conducted. A total of 77 articles met the inclusion criteria. The included articles were analyzed qualitatively, with an integrative approach.

**Results:**

The included studies demonstrated a variety of gendered norms about men's and women's experience and expression of pain, their identity, lifestyle, and coping style. Gender bias in pain treatment was identified, as part of the patient-provider encounter and the professional's treatment decisions. It was discussed how gendered norms are consolidated by hegemonic masculinity and andronormativity.

**Conclusions:**

Awareness about gendered norms is important, both in research and clinical practice, in order to counteract gender bias in health care and to support health-care professionals in providing more equitable care that is more capable to meet the need of all patients, men and women.

## 1. Introduction

Pain is a symptom in a wide range of medical conditions and can have a significant impact on a person's quality of life, general functioning, and employment status [1]. Women dominate most diagnoses related to chronic pain [1–4], and research has consistently shown differences between the sexes, like the perception, description and expression of pain, the use of coping strategies, and the benefit of different treatments [2, 5–7]. There are convincing findings that biological differences contribute to the observed sex differences [2, 3, 7]. Genetic factors, as well as hormonal factors, act as sex-specific pain mediators [2, 3, 5, 8–10]. Studies have found that women's pain responses are affected by menstrual cycle, pregnancy, and oral conceptive use [2, 3, 5, 8–10], which confirms that hormones are related to pain response. In addition, the response to opioid receptor antagonists may generate a difference between men's and women's experiences of pain [2, 3, 5, 8–10]. However, more research is needed to fully understand the underlying biological mechanisms [2, 3, 5, 8–10].

Even psychosocial factors have been uplifted as explanations for sex differences. Pain is, by definition, always subjective [11]. Pain scales, widely used to assess a person's pain in research and clinical practice [2, 12, 13], measure pain report, which, in turn, can be influenced by social factors, like gender. From an early age, boys and girls are socialized along gender norms for how to respond to pain. Myers et al. suggested that boys and men are taught to be tough, tolerate pain, and sustain painful experiences, while girls and women are socialized to be sensitive, careful, and to verbalize discomfort [14]. Sex-related expectations about pain perception influence pain responses [7]. In experimental settings, participants who scored high on masculinity showed higher pain tolerance. Participants who scored high on femininity showed higher pain sensitivity [15]. In a cold pressure task, women showed lower pain threshold and tolerance compared to men. When the condition was changed so that men and women were given the same tolerance expectations prior to the task (“the typical man/woman lasts 30 seconds”), there were no longer any differences between men and women regarding pain threshold, tolerance, and pain ratings [16].

Consequently, gender role expectations influence perception and report of pain. However, it has to be further examined to what degree sex and gender role expectations, respectively, and together influence pain ratings [17].

Neither sex nor gender alone can account for observed pain differences between men and women [2, 5, 6, 17]. The need to include both sex and gender in pain research, and to separate these concepts correctly, has been argued critically [2, 18, 19]. However, it is difficult to dissociate sex and gender—biological, psychological and social differences between men and women with respect to pain—as these differences are interrelated [2, 3, 10].

Given the still unexplained differences between men and women in pain, it is relevant to look for other explanatory factors. Of interest for such a scope is potential influence of gender norms. Gender norms are culture-based and express expectations about men's and women's interests, behaviors, and choices in life [19, 20]. Gender norms also express male power dominance where men and women are regarded as inherently different (separation), and male values are usually seen as more favorable than female attributes (dominance) [20]. Gender norms are reflected in health care. They can be held by patients, researchers, and clinicians and can lead to gender bias, medically unmotivated differences in the treatment of men and women [3, 18]. In recent years, a variety of examples have been presented in which men and women have been treated differently for the same diseases, medically unmotivated, across a wide range of medical fields including psoriasis [21], neck pain [22], heart disease [23], and polypharmacy [24]. Pain, especially chronic pain, is a field in medicine and medical care that has been pointed out to be affected by gender bias [6, 10, 13]. However, there is a lack of knowledge about how gender bias manifest in pain treatment, especially in clinical practice and in the patient-provider encounter [2, 3, 25, 26].

Chronic pain is common in all western societies [1, 4], challenging both health care and working life. In a large population-based European telephone survey, 19 percent of the participants reported moderate to severe pain, defined as lasting 6 months or more and experienced several times during the week prior to the interview [1]. The results showed that chronic pain had a pervasive impact on activities of daily life, employment status, and emotional status [1]. Patients with chronic pain occupy 20–40 percent of all consultations in Swedish primary health care [27]. Another Swedish study calculated that the mean total cost (direct and societal cost) per patient with chronic pain, per year, was 6400 EUR [28]. Given the amount of people suffering from pain and the dominance of women with pain diagnoses, it should be of great value to review, analyze, and compile research on gender norms and gender bias in pain treatment, as a tool to increase health-care providers' consciousness about potential gender bias and thereby improve equity in health and the treatment of all pain patients. This review will contribute with knowledge from different scientific fields, and with a theory-guided categorization and analysis of the literature reviewed.

## 2. Theoretical Framework

As evidence-based medicine is a cornerstone in health care, professionals need to apply research results in their daily work [29]; and their medical decisions should be based on current best evidence [29]. However, there are pitfalls, even in current research, like the inadequate generalization of results to a greater population than studied. One example is research on men as a basis for treatment of both men and women. Gender-blindness, the “nonawareness of the fact that a great deal of knowledge is based on research performed in men” [30], has been identified as an obstacle for gender equity in health care. As late as in 2007, the Sex, Gender and Pain Special Interest Group of the International Association for the Study of Pain stated that females are underrepresented in animal and human studies and recommended that “both constructs (sex and gender) should be examined when possible in order to understand their relative contribution to differences in pain between men and women” (p. 27) [3]. Without sufficient consciousness about sex and gender-biased research, it has been common to neutralize both patients and professionals [31]. Diagnostics and treatments evolved on men were announced as diagnostics and treatments for patients, including men and women. Despite regulations dictating the inclusion of men and women in medical research, gender-blind attitudes can still be observed. Hølge–Hazelton and Malterud suggested, “A notion of gender neutrality is still alive in the medical culture, suggesting that gender issues are not relevant within this field” (p. 139) [31]. Gender-blindness can lead to women's needs being overlooked, as seen in coronary heart diseases [23], but can also lead to that men's needs are failed to notice, as seen in under-diagnosed depression in men [32].

The term hegemonic masculinity describes a pattern of masculine attributes, behaviors, and practices which are constructed as the prevailing and idealized norm and against which both men and women are evaluated [33]. Hegemonic masculinity is practiced individually and structurally, is built on consensus within a social environment, and can change over time [33]. It expresses a dominance of men over women and over other men that do not live up to idealized norms like physical strength, technical competence, autonomy, and self-reliance [33]. Regarding pain patients, masculine attributes like strength, endurance, and stoicism are valued higher than feminine attributes like sensitivity and to express discomfort [6]. Even if the concept of hegemonic masculinity has been further developed and its complexity has been underlined [33], it is still used to explain dominant relations between men and women but also among men, in general [33] and in health care [6].

Even the concept of andronormativity has been discussed and applied to health care. Andronormativity implies that masculinity and male values are regarded as normal in medicine to such an extent that femininity and female values are invisible and need to be highlighted in order to be recognized [31]. Andronormativity has consequences for which conditions are prioritized or downprioritized in research and health care and may be reflected in status hierarchies of diagnoses [34]. Album and Westin showed that women-dominated conditions like fibromyalgia and anxiety neurosis were rated as the least prestigious among 38 diseases [35]. Andronormativity has also consequences for how male behavior is seen as normal in conditions that affect both men and women. Men and women with angina symptoms often express different pain locations [36]. Even though angina is common in both men and women, it has been shown that women's pain has been referred to as atypical, which in this context means not like men's pain, positioning men's pain as the norm [36].

The purpose of this study is to review literature from medical, behavioral, and social sciences on (i) gendered norms about men and women with pain, (ii) gendered norms about how men and women with pain cope with their daily life, and (iii) gender bias in the treatment of pain including both the patient-provider encounter and professional treatment decisions. However, the aim of this study is not to prove if gendered norms in health care exist—which earlier research already has shown [2, 3, 13]—but to collect and analyze gendered norms and gender bias as described in pain literature and deepen the knowledge about them. The second purpose is to analyze the findings in relation to concepts of hegemonic masculinity and andronormativity in the health care, as a general driving force of gender constructions in Western societies.

## 3. Method

In this review, sex is defined as a biological construct, in terms of differences between men and women concerning anatomy, physiology, genes, and hormones. Gender is defined as “a social construct regarding culture-bound conventions, roles, and behaviors for, as well as relations between and among, women and men and boys and girls” (p 653) [18]. Gender norms concern behaviors which are generally considered to be appropriate, desirable, and “normal” for men or for women [19]. In this review, we also use the term gendered norms. In different situations and areas in daily life, different reactions and behaviors of men and women are expected. Thus, norms about leisure activities, reactions to life events, social relations and so on are gendered and go along with different expectations on men and women, which in turn risks to consolidate the dichotomous construction of gender.

This study was designed as a theory-guided review, with the purpose to collect knowledge generated through diverse methodologies within different scientific fields. To integrate and analyze knowledge from different scientific fields, with data from empirical and theoretical literature, has been described as a way to extend existing knowledge into new insights and new holistic concepts [37–39]. Knowledge about gender norms in pain treatment can be found in the medical, behavioral, and social sciences, generated by quantitative and qualitative studies, theory development, systematical reviews, and so on. This review was theory-guided with a preunderstanding that gendered norms exist and influence the patient-provider relation and treatment decisions. However, first after the categorization of the reviewed studies, hegemonic masculinity and andronormativity were found as adequate theoretical concepts for a deeper analysis of the results.

### 3.1. Search Methods

A literature search was conducted using the following databases: PsycINFO, CINAHL, and PubMed. These databases were chosen in order to capture a broad spectrum of research from the medical, behavioral, and social sciences. The searches were limited to studies comprising human research subjects, articles written in English and published between January 2000 and April 2015. The following search terms were used, as text terms, applied to the whole article: *chronic pain* and *femininity*, *chronic pain* and *masculinity*, *chronic pain* and *gender bias*, *chronic pain* and *gender stereotypes*, and *chronic pain* and *gender roles*. The selection of search terms was theory-guided. That means, we did not review all literature about pain to examine if gender norms exist, but searched for articles that described them. The search rendered 688 articles; 175 duplicates were removed, and the other articles' title and abstract were read. The literature search was supplemented by a manual search, where relevant articles were retrieved from back references, citations, and a directed Google Scholar search. Even though the term gender roles was used, no study concerning transgender or other gender identities came up.

It has been argued that it is common practice to use the word “gender” when “sex” is meant, in health-care research in general [40], as well as in pain research in particular [6, 41]. As a consequence, articles were removed when they clearly intended to examine sex differences despite using the word “gender.” Articles were also excluded when they did not relate to health-care services. Based on the criteria outlined above, 77 articles were selected for inclusion in the review; an overview of these articles is shown in Table 1.

### 3.2. Data Analysis

The material was sorted into three theoretical categories corresponding to the research questions and thereby providing the study with a conceptual framework [42]. The theoretical categories were gendered norms about men and women with pain, gendered norms about how men and women cope with pain, and gender bias in the treatment of pain. The material in each theoretical category was further analyzed and coded into substantive categories which were more descriptive and closer to the categorized data [42]. With the amount of data in this review, it was particularly important to maintain a formal framework while at the same time allowing for new ideas and the identification of new relations between results. This was possible via the classification in both theoretical and substantive categories [42].

The theoretical and substantive categories are summarized in Table 2. The first author conceptualized the theoretical and substantial categories and discussed them with the other authors until consensus had been reached. The review only used results from the studies included in the analysis. However, there is one exception (under Inexplicable—Unfit), also marked in the text, where definitions used in different studies were analyzed. Those definitions were usually not rendered as results but used throughout the articles.

During analysis, different patterns emerged which were further analyzed through the application of two concepts: hegemonic masculinity and andronormativity. This part of the review is outlined in the discussion section.

## 4. Results

The selected 77 articles were published in 39 different journals, with studies published in journals specialized in pain dominating (32 articles). Different kinds of research design were represented, including quantitative and qualitative studies, reviews, and articles on theory development. However, only one of the studies with a qualitative design was published in a journal specializing in the field of pain. The distribution of the included articles over the 15 years varied between one and 12 articles/year, without any notifiable trend over time. All articles were conducted in high income countries—the United States, Canada, Western Europe, Australia, New Zealand, and Japan. Diagnoses that were examined included back pain (14), neck pain (5), musculoskeletal pain (9), fibromyalgia syndrome (5), osteoporosis (3), rheumatoid arthritis (2), ankylosing spondylitis, and headache. An overview over the included articles is presented in Table 1.

The results were organized according to the three theoretical categories (also the research questions of this review) and their related substantive categories. An overview of all categories and corresponding articles is presented in Table 2.

### 4.1. Gendered Norms about Men and Women with Pain

The studies reviewed showed a variety of gendered norms on how men and women experience and express pain and about patients with medically inexplicable pain conditions, such as fibromyalgia syndrome.

### 4.1.1. Stoic Men

In the studies reviewed, a clear pattern appeared, where men were presented as being stoic [6, 8–10, 13, 14, 43–50], tolerating pain [2, 13, 14, 16, 41, 45–47, 49, 51–53], denying pain [8, 45–47, 51, 53, 54], and taking health risks even when they lead to increased pain [54–56]. Further, men were described as being autonomous [43–46, 48, 57], in control [43, 45, 46, 48, 50, 57, 58], avoiding seeking health care [5, 8, 13, 44, 45, 54, 55], not talking about pain [2, 8, 13, 14, 41, 45, 59], and avoiding talking about the possible relation between pain and psychic well-being [13, 43, 45, 46, 59]. One study interviewed male physicians about men seeking medical help: “All participants attributed men's lack of contact with health services to their need to appear ‘brave and manly' which making them reluctant to admit weakness” (p. 706) [44]. In a British study about men's view on masculinity and help-seeking, one participant said, “You don't like to make a fuss because it's a macho thing just to say you're being the strong and silent type … You'll endure it, you can take it. So if there is something wrong you won't talk to anyone about it. You have to be bed-ridden or half dead before you'll go (to the doctor's)” (p. 508) [45]. The description of the “stoic man” was the same, whether it was given by researchers [2, 8, 14, 51–53], men with pain [43, 45, 46, 54, 55, 57, 59], or health-care professionals [13, 44]. Yet, some studies pointed out that men also can experience vulnerability, distress, and suffering [46, 48, 59, 60], sometimes combined with an unwillingness to talk about it [59, 60].

### 4.1.2. Sensitive Women—In Comparison

Unlike the descriptions of men who were independent from women, the reviewed studies described women in comparison to men. Women were pictured as more sensitive to pain [2, 9, 13, 14, 41, 47, 53, 61–63] and more willing to report pain than men [2, 5, 6, 8–10, 13, 14, 16, 25, 26, 41, 47, 51, 62–65]. It was also pointed out that it is more socially accepted for women than for men to show pain and talk about it [2, 8, 13, 14, 66]. In one study, health-care professionals gave different messages to men and women. “Be careful” messages were more often given to women, while “pain goes with heavy work” was more often given to men [67]. Some studies claimed that women, to a greater degree than men, are used to internal pain, because of menstruation and child birth [6, 8, 13, 47, 51, 68]. Some researchers connected this to the presumption that women have greater body awareness [8, 13], while others suggested that pain without an external cause is a natural characteristic of women's bodies [2, 6, 13, 47, 51, 68].

### 4.1.3. Hysterical Women

The reviewed studies showed that women with pain can be perceived as hysterical [8, 13, 69–71], emotional [13, 49], complaining [49, 72], not wanting to get better [71, 73–75], malingerers [71, 73], and fabricating the pain, as if it is all in her head [47, 49, 71, 74, 76]. Other studies showed that woman with chronic pain rather are assigned psychological than somatic causes for their pain [13, 22, 47, 49, 69–71, 74–79].

### 4.1.4. Inexplicable—Unfit

There are conditions where pain seems to be the only reported, visible, or measurable symptom. According to the reviewed studies, these conditions affect mostly women [8, 13, 53, 69–71, 75, 80–82]. The reviewed studies demonstrated that “medically unexplained” conditions often go along with an unwillingness among professionals to believe in the women's pain [8, 13, 69, 73, 77, 83]. In a Canadian study, general practitioners and specialists were interviewed about fibromyalgia patients [73]. They regarded fibromyalgia patients as malingerers, time consuming, and frustrating. Some clinicians even held the patients accountable for their pain [73].

Thirteen articles [6, 8, 22, 53, 69–71, 75, 77, 80–82, 84] in this review classified “medically unexplained” conditions in 19 different ways, for example, as pain without organic, observable, and objective symptoms [6]. The classifications are listed in Table 3. Those definitions were usually not rendered as results in the reviewed studies, but used throughout them. This is the only section of this review where other parts than rendered results of the reviewed studies were included. The definitions given showed a clear focus on the absence of something (diagnostic evidence, organic pathology and so on), rather than the presence of something, which one of the studies also discussed in detail [70]. Medically inexplicable pain was described as a challenge for research and clinical practice to handle since these conditions do not easily fit into the traditional bioscientific medical system [6, 69–71, 77, 80]. One researcher explained, “In fact, these conditions are called ‘contested illnesses' precisely because they represent a clash between biomedical knowledge and patient experience” (p. 834) [71].

The reviewed studies showed that legitimacy is crucial for pain patients [6, 49, 70–72, 74–76, 78, 83]. Women's narratives about their experiences with clinicians showed, “(…) how hard they have had to work to be taken seriously, believed, and understood in medical encounters” (p. 1038) [72]. The encounter between the woman with chronic pain and her physician has been described as a struggle of both patients [76, 78] and clinicians [73, 83].

### 4.2. Gendered Norms about How Men and Women Cope with Pain

According to the studies reviewed, pain affects men's and women's identity and lifestyle in different ways. The role of gender and gender identity as a relevant factor for identity, lifestyle, and coping strategies in pain patients was illustrated by the reviewed studies.

### 4.2.1. Men's Gender Identity in Jeopardy

In summary, the reviewed studies meant that chronic pain did not alter men's identity [45, 46, 54, 57, 58]. Men with pain also strived to continue to live a normal life [43, 48, 54, 56, 58, 59, 85]. However, one study pointed out that the alteration of self-identity is common for both men and women with impairments [49]. Even if chronic pain per se did not seem to affect men's identity in general, there seemed to be a connection between chronic pain and men's gender identity. Men diagnosed with pain conditions that are predominant in women or suffering from chronic pain, which was perceived as feminine, described their suffering from pain as a threat to their sense of masculinity [43, 48, 54–56, 59, 60, 85] leading to feelings of frustration [43, 54, 59, 85], irritation [48, 59, 85], shame [48, 54, 56], and grief [59, 60]. In a Danish study, a man with ankylosing spondylitis expressed this as “…a bloody dent in the masculinity that I can't lift my wife with one arm, and my two children with the other” (p. 35) [85]. Further, men with chronic pain were perceived by others—both laypeople and nurses—as being less masculine and more feminine than the typical man [50]. The same study also showed that women with pain were perceived as less feminine and more masculine than the typical woman and that men and women with chronic pain were considered to be more alike than the typical man and woman [50].

### 4.2.2. The Strong Body

The reviewed studies showed that paid work was important for men and that the role of the “breadwinner” was linked to their sense of masculinity [13, 46, 48, 49, 58, 59, 79, 85]. They also displayed the functional physical body, including muscle strength, as a central part of men's gender identity. This was a recurring theme when men described their experiences of living with pain [43, 46, 48, 54, 56, 57, 59, 60, 85]. The importance of leisure activities, particularly sports, was highlighted [43, 46, 48, 54, 56–60, 85]. A study on multiple chronic conditions later in life stated that more than half of the participants felt that their sense of masculinity was threatened when they could not participate in sports activities to the same extent as before [43]. An interview-study with men and women with chronic pain also showed that women, but not men were expected to cut down on leisure activities [58].

### 4.2.3. Men's Approach—This Is Not Me

The studies reviewed displayed different ways how men coped with pain as a threat to their masculinity. For instance, when men recognized their diagnosis as a “women's disease”, they questioned it, ignored it, or did not talk about it [48, 54–56] and showed low compliance with physicians' advice [48, 54, 55]. In focus group interviews, men with osteoporosis described how they hid their “weakness” in public. They preferred to risk increased pain and new fractures rather than following the doctor's advice about not doing heavy lifting [54]. According to the reviewed studies, men also explained their pain with factors from outside, beyond the individual's control [46, 56, 57, 60, 68, 86]. Ahlsen et al. stated, “By focusing on forces outside the men's influence and control, such as physical damage, bad genes and the nature of their work, the men's stories are often claiming the identity of being ‘good enough'” (p. 1770) [46]. The reviewed studies also showed that men considered it to be the health care's responsibility to restore their health [56, 57, 60]. For example, “In David's story, the responsibility of the self seems to be limited to keeping up with the training program, while it ultimately seems to be the health professionals' responsibility ‘to fix the problem'” (p. 364) [57]. In another study, a man with osteoporosis was quite clear about the role of health care: “I don't want to manage the pain. I want it eliminated” (p. 537) [56].

### 4.2.4. The Female Patchwork

The studies reviewed pointed out that women's identity was influenced by pain in combination with society's expectations, which included having a paid work, being a spouse and a mother, and being responsible for household and social relations [43, 47, 49, 51, 57, 58, 71, 72, 75, 76, 80, 81, 87]. It was also mentioned that women felt responsible for and prioritized family and household [13, 43, 47, 49, 57, 58, 79, 80, 87–89] and that health-care professionals encouraged them to do so [58]. In a Swedish study, more women than men were asked questions about household and family [22]. In a study on patients with low-back pain, married women with pain continued to perform household work to the same extent as before [89]. On the other hand, another study showed that men with musculoskeletal pain did not participate in household activities to the same extent as before and often handed these duties over to their spouses. Still, they saw their family role as unproblematic [58].

### 4.2.5. Women's Approach—I Have to Learn

In the reviewed studies, women faced complex demands [47, 49, 51, 57, 58, 72, 75, 79, 80, 87] and tried to manage pain and the demands of their surroundings simultaneously [43, 49, 57, 58, 75, 76, 80, 87]. According to the reviewed studies, the fact that women face complex expectations could explain why women use more and more complex coping strategies compared to men—for example, the use of social support [2, 9, 13, 14, 51, 90]. Nevertheless, women's coping strategies were rated as less functional than men's, for example by health-care students [51, 65]. Others found that men and women probably benefit from different coping strategies. It has, for example, been shown that men, but not women, benefitted from focusing on the pain [2, 9, 10, 13, 14, 25, 91].

It has been suggested that women with chronic pain have to learn to prioritize their duties and set limits to their surrounding [43, 57, 58, 75, 76, 81, 87]. In an interview study by Werner et al., women reported that they had learned during rehabilitation to explain their reduced physical capacity to their close ones and at work and to set limits for others' expectations [75]. It has also been reported that women who failed to manage their pain and demands from outside blamed themselves, which influenced their self-esteem negatively [49, 75, 80, 87]. “Subjected to repeated experiences of not being heard, understood, or taken seriously regarding their invisible but long-lasting pain, the women could still experience doubt about themselves or feel that they were to blame” (p. 500) [75].

### 4.3. Gender Bias in the Treatment of Pain

The reviewed studies showed gender bias in the encounter, along with gender bias in prescribed medication. Differences in the treatment of men and women in these studies could not be explained by different medical needs.

### 4.3.1. Struggle for Legitimacy

In the reviewed studies, women with chronic pain frequently reported being mistrusted [57, 72–75, 78, 87] and psychologized [72, 74, 78] by their health-care providers. In a study from Finland, women wrote narratives about the process of getting back pain diagnosed [74]. The results showed that doctors did not take the women's pain seriously and that the doctors' neglectful attitude became part of the problem [74]. Gustafsson et al. examined women's experiences of a rehabilitation program; women started out with a feeling of shame based on mistrust from professionals and misunderstanding from their families and friends [87]. However, women also reported that negative encounters with health care eventually changed to the better when they, often after long time, met a physician who believed them [57, 72, 74, 87]. Tait et al. described a vicious circle. Feeling mistrusted or psychologized by health-care professionals can lead to distress. Pain, accompanied by distress, is typically attributed to psychological factors. If the patient articulates the distress, it can lead to an even greater degree of psychologization by health-care professionals [82]. Evolving a theory on women and chronic pain, Skuladottir and Halldorsdottir showed that professionals could empower women by being wise, competent, caring, and building a trustful relationship with them [81]. They could also reinforce gender norms via mistrust, disrespectful treatment, and making the women responsible for not being healthy [81].

### 4.3.2. How Do I Look (Appearances)?

The reviewed studies demonstrated that the appearance of women with chronic pain was judged by their doctors [6, 13, 49, 75, 78, 83]. Some women were mistrusted when they looked too good, as in “you can't be sick,” while others were judged as unreliable if they did not look good enough [13, 78]. “Statements like ‘You don't look ill', ‘You always look so healthy!', or ‘You are so young!' had made them feel irritated, sad, and frustrated, rather than flattered. Some of them said such statements indicated little understanding for how much pain they really had” (p. 1413) [78]. In experimental settings, the sex (and sometimes attractiveness) of the experimenter influenced participants' pain responses [2, 5, 6, 8–10, 14, 15, 51, 53, 82, 92–94]. Pain threshold or tolerance tended to be higher, or pain reporting lower, when the experimenter was of the opposite sex, more pronounced for men than women.

### 4.3.3. Differences in Medication

The search for gender bias and chronic pain generated a number of studies on pain medication given to men and women [2, 13, 22, 82, 86, 95–101]. The results of these studies showed that women, compared to men, received less and less effective pain relief [2, 13, 82, 96], less pain medication with opioids [13, 86, 95, 99, 100], and more antidepressants [2, 13, 22, 86, 97, 101] and got more mental health referrals [22, 84, 97, 101]. In some of the reviewed studies, pain management decisions were affected by the clinician's own sex, thus interacting with the patient's sex [84, 101–105].

## 5. Discussion

The purpose of this study was to review and condense literature on gendered norms about men and women with pain, gendered norms about how men and women with pain cope with their daily life, and gender bias in the treatment of pain. In the following, main findings are discussed and analyzed with theories related to the concepts andronormativity and hegemonic masculinity.

Among the main findings in this review was a distinct pattern of gendered norms described in pain literature, in line with hegemonic masculinity, that distinguished men's and women's perceptions, expressions, and coping with chronic pain. For instance, men were presented as being stoic, in control, and avoiding seeking health care [45, 46]. Women, on the other hand were presented as being more sensitive to pain and more willing to show and to report pain [62, 63], compared to men. These overall findings confirm a pattern of separation between men and women, not embedded in biological differences but gendered norms. The dichotomy between men and women has been described as a way to establish and maintain the gender order, allowing men's dominance over women [33]. That women were described in comparison to men can also be seen as a proof for andronormativity in health care, stressing that men, and health problems more often present in men, tend to be considered as the norm, while women (and other social groups outside the norm) are seen as irregularities. Since men are the norm and perceived as being “normal,” women are compared to them. Although women have more pain than men [3, 7] and dominate most chronic pain diagnoses [3, 7], they are described in comparison to men, as being deviant from the norm, even when they are in majority.

Another main finding was the pattern of andronormativity in relation to certain pain diagnoses. There are conditions where pain is the only reported symptom. Those conditions are highly dominated by women and have been described as difficult to fit in to the traditional bioscientific medical system [69, 70]. They have low status in the medical hierarchy of diagnoses [35], and women with those diagnoses are often questioned as patients [69, 83]. The concept of andronormativity implies that men and masculinity dominate health care to such an extent that women and femininity become invisible. Our results showed that symptoms in women-dominated conditions that do not fit the masculine norm actually seem to be invisible. The definitions of these conditions in the reviewed studies have focused on the absence of medically provable signs, for example, “pain in the *absence* of diagnostic evidence” or “pain *without* organic pathology.” Accordingly, those conditions were not defined in their own terms but in terms of what they lack—in relation to the predominant medical norm. Interestingly, even women with those “medically unexplained” conditions have been treated as if their illness does not exist. Our results showed that those women have been described as “malingerers” or as “if the pain is all in her head” [49, 71]. An interesting finding worthy of future elaborations is that those pain conditions, which are predominantly suffered by women, are underexplored, and portrayed as a challenge for medicine [47, 70]. It would also be interesting to further investigate if the key for change lies in the dichotomous construction of gender, which can lead to different diagnoses given to men and women, despite equal needs or in the masculine stamp of bioscientific health care, which can lead to different approaches to high- and low-status diagnoses.

Another major finding is that women's pain in the reviewed studies was psychologized [13, 72]. According to hegemonic masculinity, psychological strain is feminine coded and at the same time down-valued in comparison to somatic conditions [32]. Consequently, when their pain condition is psychologized by health-care providers, women can feel that their pain is down-valued or dismissed, which in turn can cause stress [82]. Stress cues can, in turn, lead health-care providers to take patients' pain less serious [82], thus leading to a vicious circle. As long as stress and psychological strain are feminine coded, and a hierarchy between somatic and psychological findings exists in health care, there is a risk that not only the dichotomy between men's and women's pain, but also between somatic and psychological conditions is further consolidated.

Even men with chronic pain have to deal with hegemonic masculinity in health care. Physical strength is idealized in hegemonic masculinity, in opposition to weakness [33]. Chronic pain per se is a threat to idealized masculinities as pain generally goes along with loss of muscle strength. Our results indeed showed that physical strength was central for men's gender identity, whereas weakness threatened it [54, 55], and that men with chronic pain risked to be perceived as more feminine than the typical man [50]. Imbedded in hegemonic masculinity is a competition for dominance among men, and the threat of losing masculinity is a threat of losing power [33]. Men in the reviewed studies showed different strategies, like denial and rejection, to deal with what could be described as a threat of losing masculinity ideals. An example is ignoring or questioning the diagnosis, or not following clinicians' advice [48, 54]. Another interesting finding was that men according to the reviewed studies explained their pain with factors from outside, beyond their control [46, 57]. This may be a way for men to express that pain is not a part of them and their identity and could be understood as the attempt to keep the position as a masculine man by separating the feminine coded pain from the masculine man.

A recurrent finding in the studies reviewed was women's struggle to try to handle pain and multiple demands from their surroundings simultaneously [75, 76]. Traditionally, as part of the gender order, women are responsible for their home and family and to take care of themselves. However, our results showed that an overload of responsibility for family, work, household, their pain, and their wellbeing seemed to be an obstacle for recovery for women with pain [49, 87]. Our results also showed that health-care providers considered it important that women learn to say “no” to demands from others [75]. Even if this may be thought as an attempt to lower women's overload of responsibility, it can actually increase women's responsibility [75]. This could be explained by hegemonic masculinity, where the subordinate part is expected to conform to the prevailing norm, making women responsible to solve their issue and also being responsible for the outcome. The consequences of hegemonic masculinity can increase the burden on women with chronic pain, as the reviewed studies showed.

In summary, our results confirmed a paradox, highlighted by Hoffmann and Tarzian [13]; compared to men, women have more pain, and it is more accepted for women to show pain, and more women are diagnosed with chronic pain syndromes. Yet, paradoxically, women's pain reports are taken less seriously [13, 71, 78], their pain is discounted as being psychic or nonexistent [69, 70, 72], and their medication is less adequate than treatment given to men [2, 96]. This has been described as a paradox [13] but can be explained as an expression for hegemonic masculinity and andronormativity in health care.

### 5.1. The Relation between Gendered Norms and Gender Bias

Several researchers [2, 3] have emphasized the risk of gender bias in the treatment of pain; however, studies that demonstrated objectively measurable gender bias in medical treatment were less extensive and less consistent. Subjectivity in the assessment of pain makes pain experiences and pain treatment sensitive to gender norms [2, 12]. In addition, it is also reasonable to conclude that the subjectivity makes it difficult to prove malpractice related to gender. Nevertheless, when we searched for gender bias in pain, we found studies that showed that women received less adequate pain medication and more antidepressants compared to men [86, 98]. In addition, a pattern of parallels between gendered norms and gender bias could be demonstrated in the results. For example, gendered norms were expressed through presumptions such as “women are more emotional than men” [49, 71]. The psychologizing of women's pain [13, 70] reflects this norm, and that antidepressants are more often described to women compared to men [22, 97] could be a consequence of it.

### 5.2. Consequences of Gendered Norms in Health Care

The notion of men and women as separate and different in manners and needs is problematic [106], as it can consolidate gendered norms, which in turn can lead to individual needs being overlooked [106]. Health is constituted within a wide range of gender-related experiences [106]. The patient-provider relation is one domain for constitution, reinforcement, or challenge of gendered norms, where andronormativity and hegemonic masculinity can cause health-care providers to treat men and women based on gendered norms rather than individual needs. For instance, gender norms like “men need to be physically strong” [43, 54, 58] can lead to the presumption that active leisure time is more important for men than for women, which in turn can lead health-care professionals to recommend men, but not women, to continue with sport activities despite their pain [54, 85]. Or, as another example, if women are seen as the primary care giver and responsible for family and household [49, 58, 71, 80], this can lead professionals to recommend women, but not men, to prioritize family above work and leisure time [22, 58]. Increased awareness of gendered norms and potential gender bias is a prerequisite to counter gender bias in health care [20]. There is a power imbalance between men and women, and many (though not all) gender biases are to women's disadvantage [20]. However, both men and women are restricted by gendered expectations, and both men and women profit from more equitable care [3, 20].

### 5.3. Methodological Considerations

This review was theory-guided with a preunderstanding that gendered norms exist in health care, which has influenced the selection of our search terms. Our directed literature search might be criticized as it potentially excluded studies that did not find/report gender differences. However, the aim of this study was not to prove if gendered norms in health care exist, which earlier research already has shown [2, 3, 13], but to collect and analyze gendered norms and gender bias as described in pain literature and deepen the knowledge about them. Our results support the idea that there is hegemonic masculinity and andronormativity in health care, and several patterns of gendered norms and consequences thereof could be explained by hegemonic masculinity and andronormativity. It might be important to underline that these theoretical concepts were not chosen in advance but found applicable after the categorization and analysis of the reviewed studies.

Another concern addresses the large number of included studies, providing a risk for fragmentation and selective interpretation of their content. This was balanced by the coding in three distinct and clearly defined theoretical categories, which provided a tight framework for the selection of relevant material [39, 42]. All authors discussed and agreed also on all categories. The descriptive basis of the substantive categories allowed to capture different patterns. There might be other patterns to be found in the reviewed studies. However, our findings were consistent throughout the reviewed studies and provided new insights, which should be further examined in both qualitative and quantitative studies.

A common dilemma in gender research involves how to create awareness about stereotypes without confirming or reinforcing them [40]. The purpose of this study was to challenge stereotypes about men and women, not to emphasize the differences. Gender norms are not the only norms that influence treatment decisions and patient-provider relations in health care. For instance, presumptions on age, race, and educational level have an impact on pain and intersect with each other and with gender [3, 97, 102], which is an important field for further elaboration.

## 6. Conclusions

Gendered norms about men and women with pain, present in research from different scientific fiends, illustrate prevailing hegemonic masculinity and andronormativity in health care. Yet, the notion of gender is a construction and can be changed. Awareness about gendered norms and that they can lead to a consolidation of the dichotomous depiction of men and women is important, both in research and clinical practice, in order to counteract gender bias in health care and to support health-care professionals in providing more equitable care.

## Figures and Tables

**Table 1:** Included articles, sorted by type of journal and type of study design.

	Number of articles	Included articles
*Type of journal*		
Journals specialized in pain	32	Fillingim et al. [2], Bernardes et al. [6], Racine et al. [10], Myers et al. [14], Alabas et al. [15], Robinson et al. [16], Robinson and Wise [26], Robinson et al. [41], Dao and Leresche [47], Bernardes and Lima [50], Pool et al. [52], Robinson et al. [61], Martel et al. [62], Martel et al. [63], Robinson and Wise [64], Stutts et al. [65], Hobara [66], Bernardes et al. [68], Hayes et al. [73], LaChapelle et al. [76], Tait et al. [82], Frantsve and Kerns [83], Bernardes et al. [84], Racine et al. [86], Keogh and Herdenfeldt [91], Kállai et al. [93], Aslaksen et al. [94], Green et al. [96], Hirsh et al. [97], Hirsh et al. [101], Weisse et al. [102], Marquié et al. [105]
Social sciences	6	O'brien et al. [45], Barker [71], Werner et al. [72], Lillrank [74], Werner and Malterud [78], McClelland and McCubbin [90]
Psychology	6	Hale et al. [44], Jarrett [53], Grace [70], Bernardes and Lima [77], Pujal and Mora [80], Sheffer et al. [89]
Rehabilitation	5	Ahlsen et al. [46], Côté and Coutu [49], Ahlsen et al. [57], Gustafsson et al. [87], Boonstra et al. [88]
Musculoskeletal care	4	Leresche [25], Lack et al. [48], Madsen et al. [85], Fillingim et al. [98]
Qualitative health studies	3	Paulson et al. [59], Werner et al. [75], Skuladottir and Halldorsdottir [81]
Men's health	2	Keogh [9], Nielsen et al. [54]
Women's health	2	Hamberg et al. [22], Katz et al. [69]
Elderly	3	Clarke and Bennett [43], Solimeo [55], Solimeo et al. [56]
Emergency medicine	3	Lord et al. [95], Chen et al. [99], Michael et al. [100]
Caring sciences	2	Stenberg et al. [67], Damsgård et al. [79]
Internal medicine	2	Barsky et al. [8], Weisse et al. [104]
Other medical	3	Hurley and Adams [5], Smitherman and Ward [51], Criste [103]
Other	4	Hoffmann and Tarzian [13], Kvam et al. [58], Ahlsen et al. [60], Gijsbers and Nicholson [92]

*Type of study design*		
Quantitative	33	Robinson et al. [16], Hamberg et al. [22], Robinson and Wise [26], Robinson et al. [41], Bernardes and Lima [50], Pool et al. [52], Robinson et al. [61], Martel et al. [62], Martel et al. [63], Robinson and Wise [64], Stutts et al. [65], Hobara [66], Bernardes and Lima [77], Bernardes et al. [84], Racine et al. [86], Boonstra et al. [88], Sheffer et al. [89], McClelland and McCubbin [90], Keogh and Herdenfeldt [91], Gijsbers and Nicholson [92], Kállai et al. [93], Aslaksen et al. [94], Lord et al. [95], Green et al. [96], Hirsh et al. [97], Fillingim et al. [98], Chen et al. [99], Michael et al. [100], Hirsh et al. [101], Weisse et al. [102], Criste [103], Weisse et al. [104], Marquié et al. [105]
Qualitative	21	Clarke and Bennett [43], Hale et al. [44], O'brien et al. [45], Ahlsen et al. [46], Lack et al. [48], Nielsen et al. [54], Solimeo et al. [56], Ahlsen et al. [57], Kvam et al. [58], Paulson et al. [59], Ahlsen et al. [60], Stenberg et al. [67], Barker [71], Werner et al. [72], Lillrank [74], Werner et al. [75], LaChapelle et al. [76], Werner and Malterud [78], Damsgård et al. [79], Madsen et al. [85], Gustafsson et al. [87]
Mixed methods	2	Bernardes et al. [68], Hayes et al. [73]
Review	15	Fillingim et al. [2], Hurley and Adams [5], Bernardes et al. [6], Barsky et al. [8], Keogh [9], Racine et al. [10], Hoffmann and Tarzian [13], Myers et al. [14], Alabas et al. [15], Leresche [25], Dao and Leresche [47], Côté and Coutu [49], Smitherman and Ward [51], Tait et al. [82], Frantsve and Kerns [83]
Commentary	3	Solimeo [55], Katz et al. [69], Grace [70]
Theory development	2	Pujal and Mora [80], Skuladottir and Halldorsdottir [81]
Feature	1	Jarrett [53]

**Table 2:** Theoretical categories, substantive categories, and referenced articles; 77 articles reviewed.

Theoretical category	Substantive category	References
Gendered norms about men and women with pain	Stoic men	Fillingim et al. [2], Hurley and Adams [5], Bernardes et al. [6], Barsky et al. [8], Keogh [9], Racine et al. [10], Hoffmann and Tarzian [13], Myers et al. [14], Robinson et al. [16], Robinson et al. [41], Clarke and Bennett [43], Hale et al. [44], O'brien et al. [45], Ahlsen et al. [46], Dao and Leresche [47], Lack et al. [48], Côté and Coutu [49], Bernardes and Lima [50], Smitherman and Ward [51], Pool et al. [52], Jarrett [53], Nielsen et al. [54], Solimeo [55], Solimeo et al. [56], Ahlsen et al. [57], Kvam et al. [58], Paulson et al. [59], Ahlsen et al. [60]
	Sensitive women—in comparison	Fillingim et al. [2], Hurley and Adams [5], Bernardes et al. [6], Barsky et al. [8], Keogh [9], Racine et al. [10], Hoffmann and Tarzian [13], Myers et al. [14], Robinson et al. [16], Leresche [25], Robinson and Wise [26], Robinson et al. [41], Dao and Leresche [47], Smitherman and Ward [51], Jarrett [53], Robinson et al. [61], Martel et al. [62], Martel et al. [63], Robinson and Wise [64], Stutts et al. [65], Hobara [66], Stenberg et al. [67], Bernardes et al. [68]
	Hysterical women	Barsky et al. [8], Hoffmann and Tarzian [13], Hamberg et al. [22], Dao and Leresche [47], Côté and Coutu [49], Katz et al. [69], Grace [70], Barker [71], Werner et al. [72], Hayes et al. [73], Lillrank [74], Werner et al. [75], LaChapelle et al. [76], Bernardes and Lima [77], Werner and Malterud [78], Damsgård et al. [79]
	Inexplicable—unfit	Bernardes et al. [6], Barsky et al. [8], Hoffmann and Tarzian [13], Hamberg et al. [22], Côté and Coutu [49], Jarrett [53], Katz et al. [69], Grace [70], Barker [71], Werner et al. [72], Hayes et al. [73], Lillrank [74], Werner et al. [75], LaChapelle et al. [76], Bernardes and Lima [77], Werner and Malterud [78], Pujal and Mora [80], Skuladottir and Halldorsdottir [81], Tait et al. [82], Frantsve and Kerns [83], Bernardes et al. [84]

Gendered norms about how men and women cope with pain	Men's gender identity in jeopardy	Clarke and Bennett [43], O'brien et al. [45], Ahlsen et al. [46], Lack et al. [48], Côté and Coutu [49], Bernardes and Lima [50], Nielsen et al. [54], Solimeo [55], Solimeo et al. [56], Ahlsen et al. [57], Kvam et al. [58], Paulson et al. [59], Ahlsen et al. [60], Madsen et al. [85]
	The strong body	Hoffmann and Tarzian [13], Clarke and Bennett [43], Ahlsen et al. [46], Lack et al. [48], Côté and Coutu [49], Nielsen et al. [54], Solimeo et al. [56], Ahlsen et al. [57], Kvam et al. [58], Paulson et al. [59], Ahlsen et al. [60], Damsgård et al. [79], Madsen et al. [85]
	Men's approach—this is not me	Ahlsen et al. [46], Lack et al. [48], Nielsen et al. [54], Solimeo [55], Solimeo et al. [56], Ahlsen et al. [57], Ahlsen et al. [60], Bernardes et al. [68], Racine et al. [86]
	The female patchwork	Hoffmann and Tarzian [13], Hamberg et al. [22], Clarke and Bennett [43], Dao and Leresche [47], Côté and Coutu [49], Smitherman and Ward [51], Ahlsen et al. [57], Kvam et al. [58], Barker [71], Werner et al. [72], Werner et al. [75], LaChapelle et al. [76], Damsgård et al. [79], Pujal and Mora [80], Skuladottir and Halldorsdottir [81], Gustafsson et al. [87], Boonstra et al. [88], Sheffer et al. [89]
	Women's approach—I have to learn	Fillingim et al. [2], Keogh [9], Racine et al. [10], Hoffmann and Tarzian [13], Myers et al. [14], Leresche [25], Clarke and Bennett [43], Dao and Leresche [47], Côté and Coutu [49], Smitherman and Ward [51], Ahlsen et al. [57], Kvam et al. [58], Stutts et al. [65], Werner et al. [72], Werner et al. [75], LaChapelle et al. [76], Damsgård et al. [79], Pujal and Mora [80], Skuladottir and Halldorsdottir [81], Gustafsson et al. [87], McClelland and McCubbin [90], Keogh and Herdenfeldt [91]

Gender bias in the treatment of pain	Struggle for legitimacy	Ahlsen et al. [57], Werner et al. [72], Hayes et al. [73], Lillrank [74], Werner et al. [75], Werner and Malterud [78], Skuladottir and Halldorsdottir [81], Tait et al. [82], Gustafsson et al. [87]
	How do I look? (appearances)	Fillingim et al. [2], Hurley and Adams [5], Bernardes et al. [6], Barsky et al. [8], Keogh [9], Racine et al. [10], Hoffmann and Tarzian [13], Myers et al. [14], Alabas et al. [15], Côté and Coutu [49], Smitherman and Ward [51], Jarrett [53], Werner et al. [75], Werner and Malterud [78], Tait et al. [82], Frantsve and Kerns [83], Gijsbers and Nicholson [92], Kállai et al. [93], Aslaksen et al. [94]
	Differences in medication	Fillingim et al. [2], Hoffmann and Tarzian [13], Hamberg et al. [22], Tait et al. [82], Bernardes et al. [84], Racine et al. [86], Lord et al. [95], Green et al. [96], Hirsh et al. [97], Fillingim et al. [98], Chen et al. [99], Michael et al. [100], Hirsh et al. [101], Weisse et al. [102], Criste [103], Weisse et al. [104], Marquié et al. [105]

**Table 3:** Medically inexplicable pain conditions. List of classification terms and references among 77 articles reviewed.

Classification	Reference
Pain without organic, observable, and objective symptoms	Bernardes et al. [6]
Pain without obvious cause	Bernardes et al. [6]
Medically unexplained symptoms	Barsky et al. [8]
Diagnoses of nonspecific symptoms and signs	Hamberg et al. [22]
Nonspecific symptom diagnoses	Hamberg et al. [22]
Chronic pain with unclear cause	Jarrett [53]
Disorders in the absence of organic lesions	Katz et al. [69]
Conditions, typically chronic, where no pathology can be identified in biomedical terms on diagnostic investigation	Grace [70]
Somatically experienced health problems that have no corresponding pathology	Grace [70]
Pain without objectively verifiable evidence of a somatic disease	Grace [70]
Pain without organic pathology	Grace [70]
A cluster of common and troubling symptoms (e.g., pain, fatigue, and mood irregularities) that are not attributable to any organic abnormality	Barker [71]
Disorders with a lack of conventional biomedical evidence	Barker [71]
Medically unexplained disorders	Werner et al. [75]
Pain in the absence of “objective” diagnostic evidence of pathology	Bernardes and Lima [77]
Chronic pain without organic cause	Pujal and Mora [80]
Chronic nonmalignant pain	Skuladottir and Halldorsdottir [81]
“Medically unexplained symptoms”	Tait et al. [82]
Pain in the absence of diagnostic evidence of pathology	Bernardes et al. [84]
